# Ectosymbionts alter spontaneous responses to the Earth’s magnetic field in a crustacean

**DOI:** 10.1038/s41598-018-38404-7

**Published:** 2019-02-28

**Authors:** Lukas Landler, James Skelton, Michael S. Painter, Paul W. Youmans, Rachel Muheim, Robert P. Creed, Bryan L. Brown, John B. Phillips

**Affiliations:** 1 Department of Biological Sciences, Virginia Tech, Blacksburg, VA 24061 USA; 2 School of Forest Resources and Conservation, University of Florida, Gainesville, Florida 32603 USA; 3 Czech University of Life Sciences Prague, Department of Game Management and Wildlife Biology, Kamýcká 129, CZ - 165 21 Praha 6, Suchdol, Czech Republic; 4 Department of Biology, Lund University, Lund, SE-221 00 Sweden; 5 Department of Biology, Appalachian State University, Boone, NC 28608 USA

## Abstract

Magnetic sensing is used to structure every-day, non-migratory behaviours in many animals. We show that crayfish exhibit robust spontaneous magnetic alignment responses. These magnetic behaviours are altered by interactions with Branchiobdellidan worms, which are obligate ectosymbionts. Branchiobdellidan worms have previously been shown to have positive effects on host growth when present at moderate densities, and negative effects at relatively high densities. Here we show that crayfish with moderate densities of symbionts aligned bimodally along the magnetic northeast-southwest axis, similar to passive magnetic alignment responses observed across a range of stationary vertebrates. In contrast, crayfish with high symbiont densities failed to exhibit consistent alignment relative to the magnetic field. Crayfish without symbionts shifted exhibited quadramodal magnetic alignment and were more active. These behavioural changes suggest a change in the organization of spatial behaviour with increasing ectosymbiont densities. We propose that the increased activity and a switch to quadramodal magnetic alignment may be associated with the use of systematic search strategies. Such a strategy could increase contact-rates with conspecifics in order to replenish the beneficial ectosymbionts that only disperse between hosts during direct contact. Our results demonstrate that crayfish perceive and respond to magnetic fields, and that symbionts influence magnetically structured spatial behaviour of their hosts.

## Introduction

The available evidence suggests that sensitivity to the geomagnetic field is widespread among motile animals and plays a fundamental role in organizing spatial behaviour^1^. Representatives from all vertebrate classes^2–5^ and invertebrates such as molluscs and arthropods^6^ use the Earth’s magnetic field to process directional and spatial information across multiple spatial scales. Internal and external symbionts have previously been shown to alter non-magnetic spatial behaviour of animals, either as an incidental by-product of infection^7^, or as a form of host manipulation that may increase symbiont fitness, often at a cost to host fitness^8^. Since magnetic perception appears to play an integral role in spatial behaviours, could it be possible that symbionts alter magnetic field responses in animals? Most, if not all animals host a myriad of symbionts. Without consideration of how symbiotic interactions influence magnetically structured spatial behaviour, our understanding of both host responses to magnetic cues and the effects of symbionts on these host responses might remain incomplete.

Use of magnetic cues is not limited to long-range movements such as migration, but has also been proposed to serve as a global reference system helping to integrate spatial information from other sensory modalities, underlying perception of both familiar and unfamiliar surroundings^1^. For example, honeybees use the magnetic field vector as a directional reference when approaching a visual target from a fixed direction, which may simplify encoding of geometric patterns^9,10^. A similar strategy may be used by a wide variety of animals that have been shown to spontaneously align their bodies with Earth’s magnetic field, a behaviour termed spontaneous magnetic alignment (SMA)^4,11,12^. As used in this paper, SMA specifically describes a consistent, non-goal oriented and untrained alignment of the animal body axis relative to a magnetic field^4^, and may offer fitness advantages by providing a fixed (global) reference for local spatial behaviour over multiple spatial scales and/or simplify encoding of spatial features of the animal’s surroundings^1,4^.

SMA offers advantages to researchers over other forms of magnetic behaviour for experimental studies of magnetic sensing. Unlike goal-oriented movements such as homing and learned magnetic compass orientation^13–15^, studies examining SMA do not require assays that provide conditions suitable for learning a directional response, for motivating the animal to express the learned response, or for testing conditions that do not negatively reinforce the animals attempting to orient in the learned direction. Instead, SMA appears to be a default response that many animals show reliably in the absence of reinforcement, and therefore is well suited to investigate the environmental factors that influence responses to magnetic cues and ultimately to characterize the biophysical mechanism that underlies this response.

The effects of symbionts on host spatial behaviour are often the result of multiple interacting modes of selection that can be generally categorized into three types: selection acting on the host, selection acting on the symbiont, and bi-products of host-symbiont interactions. For example, fish plagued by ectoparasites will seek cleaning stations inhabited by mutualistic partners that remove harmful ectoparasites^16–18^. This example illustrates two important drivers of symbiont effects on host fitness and resultant behaviour; first, potential negative effects of ectoparasites and secondly the benefit of interacting with cleaner species that remove ectoparasites^19^. But changes in host behaviour may also reflect selection acting on symbionts to manipulate their hosts and increase symbiont transmission^8^. Examples include lancet liver flukes (*Dicrocoelium dendriticum*) that cause ants to climb blades of grass and thereby increase the likelihood of transmission to grazing animals; hairworms (*Paragordius tricuspidatus*) that alter the reaction of their insect hosts to light and water increasing the likelihood that worms will be released in the water to continue their life cycle^20^; and protists (*Toxoplasma gondii*) that cause rats to seek, rather than avoid, odours from cat predators, which are their secondary hosts^21^. Lastly, some effects of symbionts on host behaviour may be purely incidental with no apparent selective advantage for either host or symbiont. For instance, fleas change mammalian spatial behaviour by introducing distracting sensory stimuli that may alter movement in relation to resources or potential predators^7^. Regardless of causality, these examples clearly indicate that our understanding of a host’s spatial behaviour is often incomplete without consideration of its symbionts. In this study, we characterize spontaneous magnetic alignment in a population of freshwater crayfish (*Cambarus appalachiensis*) and experimentally test for the effects of external annelid symbionts on crayfish magnetic behaviour.

Astacoidean crayfish throughout the Northern Hemisphere are hosts to a monophyletic clade of obligate ectosymbiotic worms (Annelida: Branchiobdellida)^22^. We chose this system to explore symbiont effects on magnetic behaviour for four reasons. First, branchiobdellidan presence and density on a host are easily manipulated and thus the system provides an excellent experimental model to investigate the effects of symbionts on their host^22,23^. Second, branchiobdellidans have complex fitness effects on their hosts. Moderate densities of some branchiobdellidan species may increase crayfish growth and survivorship by cleaning harmful epibiotic accumulations from their host^24,25^, whereas host growth is decreased at high density^26^, at least in part due to facultative parasitism. Third, sensory input from branchiobdellidans that elicits changes in host behaviour likely reflects co-evolved feedback between host and symbiont. For instance, branchiobdellidans alter host grooming behaviours^22,27^, and this host response is adjusted with changing costs and benefits of symbiosis through host ontogeny^23^. Lastly, alteration of crayfish spatial behaviour may affect symbiont fitness by modulating transmission, because transmission of branchiobdellidans requires contact between hosts^22^.

We used the crayfish-branchiobdellidan system to examine symbiont effects on SMA under three distinct scenarios; no symbionts, beneficial (moderate) symbiont densities, and parasitic (high) symbiont densities. We conducted a baseline study using crayfish with a range of naturally occurring symbiont densities that span beneficial effects at moderate values, and negative fitness effects at high values^22^. We then verified effects of naturally occurring symbionts on magnetic alignment by experimentally manipulating symbiont density. Our results indicate that complex host-symbiont interactions modulate magnetic behaviour and that effects of symbionts on hosts and hosts on symbionts cannot be considered independently.

## Methods

### Details of experimental animals

For all experiments, crayfish (*Cambarus appalachiensis*) were collected from Sinking Creek (Virginia, USA) (37.10°, −80.48°) one day prior to testing. They were held overnight at room temperature in plastic bags filled with water from their creek and brought to the testing facility (Behavioural Testing Facility, Virginia Tech, Virginia, USA) on the day of testing. The sampled stretch of the creek has a flow direction along the NW-SE axis (310°/130°), which results in a 40°/220° land-water axis (‘Y-axis’, definition after Ferguson and Landreth^28^). The carapace length, mass and sex of all animals were determined prior to the experiments. The mean carapace length of animals used in the baseline test was 33.6 ± 3.1 mm and the mass was 14.2 ± 4.6 g. The mean carapace length for the worm manipulation experiment was 31.0 ± 3.0 mm and the mass 11.2 ± 4.0 g.

Prior to testing in the worm manipulation experiment, we manipulated the density of the branchiobdellidan *Cambarincola ingens* following published methods^26^. In short, branchiobdellidans were removed manually by forceps and held in small glass dishes of stream water. Crayfish were then submerged in a 10% MgCl_2_ hexahydrate solution for 5 min to kill any remaining worms and cocoons, and then returned individually to bags of stream water (free of additional branchiobdellidans). Crayfish were then randomly assigned to 3 treatment groups; 0, 6, and 12 *C. ingens*. Previous experiments have shown that applying 4–6 *C. ingens* per crayfish increases host growth compared to 0-worm controls, whereas applying 12 worms decreases host growth^26^. Worms were applied to the dorsal and lateral aspects of the carapace with fine tipped forceps. The 0-worm group was subjected to identical handling conditions without application of worms.

### Testing details

The animals were brought to the indoor testing set-up and were individually placed into separate radially symmetrical individual chambers (Fig. S3), which have been successfully used in previously published magnetic alignment studies^29,30^. The animals were transported in an opaque black container (13 cm by 13 cm) with a black opaque lid to prevent access to any visual cues while transporting the animal. During transport the container was rotated at least ten times to eliminate possible path integration. As described in the earlier papers 12 animals were tested in parallel. The chambers were placed in the centre of a pair of cube-surface coils^31^, surrounded by a Faraday cage to shield out high-frequency noise (see below for details regarding the magnetic stimulation). In case of the worm manipulation experiment, we randomized the order of worm treatments, balancing treatments for position in the coil and test day (i.e. every treatment was tested on each test day). Tests were excluded from analysis if thunderstorms/rain events occurred during testing which were audible inside the testing room. In three cases a crayfish died in the experimental chamber and these data were therefore excluded from further analysis. A chamber located outside the Faraday cage and identical to the experimental chambers was filled with water, and used as a reference to measure water temperature. During the baseline test, air temperature was 24.6 ± 2.2 °C, and water temperature was 21.6 ± 1.9 °C. During the worm manipulation experiments air temperature was 19.9 ± 2.5 °C and water temperature was 12.3 ± 1.4 °C. Temperature differences between experiments reflect to some extent outside temperatures at the time of testing. While there was considerable temperature difference between the experiments, temperatures were stable throughout each of the experiments.

### Magnetic field manipulations

The magnetic fields were produced by double wrapped Rubens coils^31^, producing a total field intensity of 50.99 ± 0.37 µT and inclination angle of 64.5 ± 0.7°, identical to the ambient magnetic field. The coils were used to position the earth-strength magnetic fields into one of four cardinal compass alignments (mN at tN, tE, tS, and tW). In case of the worm-density experiment animals were tested in a random sequence of all four magnetic field alignments. In the baseline experiments animals were tested in two randomly chosen perpendicular field alignments. This process allowed us to uncouple magnetic field directions from all other fixed spatial references and to separate magnetic and non-magnetic (topographic) responses (see Fig. S4 and Fig. 3 in Muheim, *et al*.^13^ for detailed description of magnetic versus topographic analyses). Pooling data from all four fields, or two perpendicular alignments either with respect to absolute (‘topographic’) north, or with respect to the alignment of magnetic north in testing made it possible to isolate the component of directional behaviour that was a response to the magnetic field, from the component that was a response to non-magnetic cues (see Fig. S4). The analysis software used to evaluate these data only recorded the axis of the crayfish body, and therefore, there is no difference between northward or southward alignment (see below for details concerning the analysis software). All experiments were conducted in a grounded Faraday cage shielding the test environment from ambient radio-frequency noise shown to disrupt magnetic orientation^32,33^. All changes in the magnetic field alignment were made from outside the Faraday cage without disturbing the animals or removing them from the testing chambers.

All animals were exposed to different magnetic field alignments for a total of four hours. The alignment of the magnetic field was changed after each hour in the worm-density experiment and after two hours in the baseline experiment. Thus, the two experimental procedures remained identical in total time the crayfish were exposed to manipulated magnetic fields.

### Video analysis

A camera located beneath the experimental arena and connected to a computer recorded the silhouette of the animals through a translucent arena floor. To determine the directional responses of the animals, we analysed 30 min out of each hour for each magnetic field treatment in the worm-density experiment, deleting the first and last 15 min, which resulted in a total of 2 h of analysed data (4 × 30 min). In the baseline experiment, we used 1 h out of the 2 h magnetic field exposure, deleting the first and last 30 min, in total analysing 2 h of directional responses (2 × 1 h). It is important to note that the time analysed was the same (2 h) in both experiments. We excluded the beginning and end of each treatment in order to reduce potential carry-over effects from one magnetic field alignment to the next and other potential disturbances that might have arisen from the experimenter entering the outer room of the testing building and switching the magnetic field conditions.

The videos were recorded in Virtual Dub, converted to one frame per second, and then were tracked and analysed using custom-programmed tracking software ‘Alignment v4’ (programmed by co-author Rachel Muheim in Matlab R2012a, The Mathworks Inc.). The tracking software initiated tracking an animal when the first movement was detected and recorded the axis of body alignment (i.e. did not distinguish anterior and posterior along the body axis). The software also calculated an activity index, which is a measure of the movement of the animals that includes lateral movements. This index provides a comprehensive measurement of overall activity but cannot be transformed into distance travelled. To compare the effects of worm treatment on overall activity, the activity of each individual animal for all four hours of video from the worm manipulation experiment was analysed.

### Standard circular statistic details and activity analysis

We calculated the mean axis of orientation of each individual crayfish relative to topographic and magnetic north by combining the axial responses of the two (baseline experiment) or four (worm-density experiment) magnetic field alignments. Based on preliminary trials (Landler *et al*., unpublished) as a reference, the expected orientation was an axial alignment response (i.e. crayfish aligning along an axis), however, the response to the worm treatments was unknown. Therefore, we also tested for quadramodal alignment (i.e. four symmetrical clusters, each separated by 90°). A Bonferroni correction was used to correct for multiple hypothesis testing. All circular statistics were conducted in Oriana 4^34^. To test for quadramodal orientation, bearings were first multiplied by a factor of four (quadrupled) and then reduced to modulo 360°, and tested for unimodal orientation^35^. For the remaining analysis, the data were treated as ‘axial’ in Oriana. Activity between treatments was compared using an ANOVA with day as a blocking factor, and group-wise differences were assessed by post hoc Tukey’s test.

To compare the results of the baseline study to the worm manipulation study, the un-manipulated symbiont densities found on crayfish during the baseline experiment were classified into two groups, low density (<5 worms) and high density (≥5 worms) based on the naturally occurring density of *C. ingens* observed on each individual immediately after testing. The moderate-density baseline class corresponds to the expected density of the 6-worm treatment, after accounting for attrition of experimentally applied worms during the first 24 hours, which is typically ~30%^23^. Likewise, the high-density class corresponds to the 12-worm treatment. Because of the naturally high prevalence of *C*. *ingens* on adult *C*. *appalachiensis* (nearly 100% at our research site), our baseline experiment did not have a 0-worm equivalent, i.e. all field-collected individuals had at least low densities of symbionts. In order to compare the two experiments we analysed the circular standard deviation in both experiments with respect to their ectosymbiont densities. Because we wanted to know how variation among indivuals would change with varying ectosymbiont densities, we calculated the circular standard deviation of the mean vectors for a moving window of 12 individuals along a gradient of ectosymbiont densities. This was done by first sorting the crayfish according to increasing ectosymbiont loads. We then calculated the circular standard deviation for the first 12 animals in the list, then shifted the frame one individual down and calculated the same statistic for the 12 consecutive animals starting from the second in the list, then the 3rd, and so on until the moving frame reached the end of the list.

## Results

**Figure 1:**
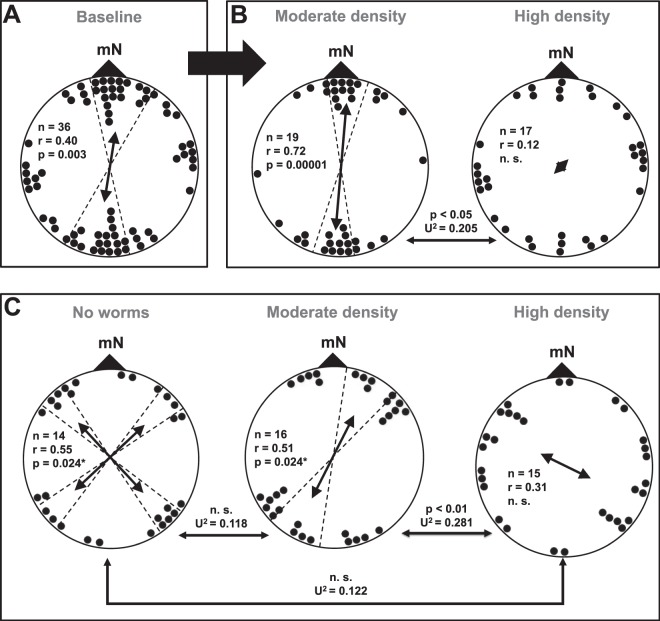
Magnetic alignment of crayfish. We tested spontaneous magnetic alignment behaviour of crayfish in a baseline assay without prior manipulation of ectosymbiont densities (**A**). We split the distribution of crayfish alignments based on symbiont densities above (high) and below (moderate) the median value of 5 worms (**B**). We also tested magnetic alignment behaviour of crayfish with manipulated worm densities at three treatment levels; no worms, moderate and high worm density (**C**). Baseline test demonstrated a significant magnetic alignment of crayfish to the magnetic NE/SW axis. Crayfish with moderate worm densities exhibited a bimodal distribution of alignments with respect to the magnetic field, whereas those with high worm densities were not magnetically aligned. Crayfish tested with three different ectosymbiont densities showed a quadramodal distribution of alignments when hosts were without worms ‘no worms’, a bimodal distribution of alignments when hosts had a moderate worm density, and a distribution of magnetic alignments that was indistinguishable from random when hosts had a high worm density. Significance of alignments was tested by the Rayleigh-test; p-value (p), sample size and the mean vector length (r) are given for each distribution. We compared distributions using the Watson U^2^-test; test statistic (U^2^) and p-values are shown above the arrows indicating the compared distributions. All p-values with an asterisk are alpha corrected, when tested for axial and bi-axial alignment. We calculated a 95% confidence interval around the mean direction confidence interval in case of significance.

In our baseline study with un-manipulated symbiont densities, the overall distribution of crayfish alignments was clustered along the NNE-SSW magnetic axis (Fig. 1A). In contrast, no clustering in the overall distribution of topographic alignment was detected (see Fig. S1A). Within the overall distribution, naturally occurring differences in symbiont density on individual crayfish explained much of the variation in crayfish spontaneous magnetic alignment (SMA) responses. At moderate densities (<5 worms per crayfish) SMA was highly significant (Fig. 1B), whereas topographic alignment was not significantly different from a random distribution (Fig. S1B). The reverse was true of crayfish with high worm densities (≥5 worms); SMA was not significantly different from a random distribution (Fig. 1B), but instead the crayfish showed a significant alignment along the topographic east-west axis (Fig. S1B). Therefore, high worm densities did not disrupt the use of alternative (non-magnetic) cues, but rather changed the relative utility or salience of magnetic versus non-magnetic cues.

**Figure 2:**
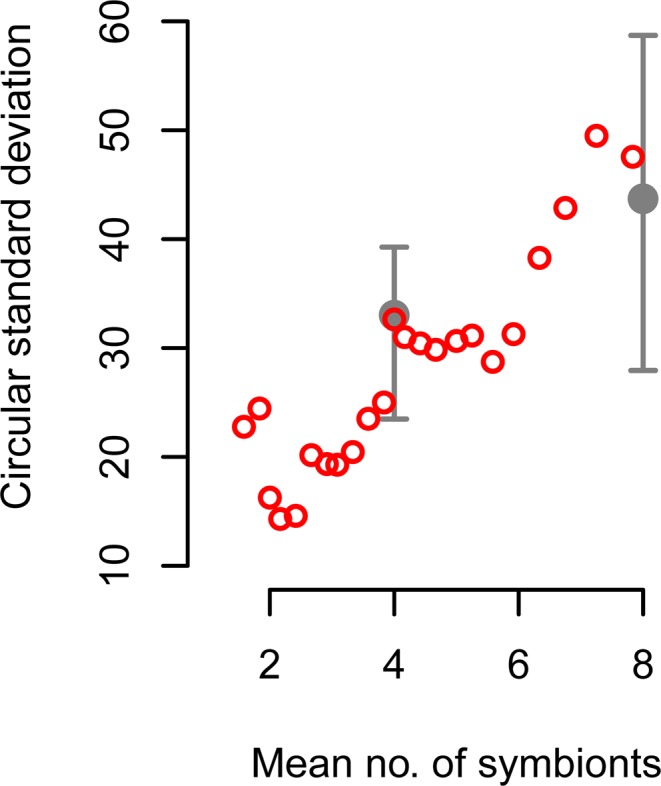
Variability in magnetic response as a function of symbiont density. Comparison of the relationship between variability of crayfish magnetic orientation (circular standard deviation of mean orientation among individuals) and the number of symbionts during the baseline study with natural symbiont density (red circles) and experimentally manipulated symbiont densities (grey circles). Variability in the distribution of responses to the magnetic field increased with increasing symbiont density in both the baseline and worm-density manipulation experiments. Variability in baseline data was calculated as the circular standard deviation of orientation direction across a moving window of twelve individuals sorted by increasing observed natural symbiont density. Mean number of symbionts is the average number of *C. ingens* observed on the moving window of twelve individuals. Variability for manipulated symbiont density is shown as the circular standard deviation for each treatment level. Error bars show 90% confidence interval based on bootstrapped values from 10,000 resamplings without replacement. Treatment levels were adjusted to reflect symbiont attrition prior to testing. The no-worm treatment is not shown; since condition was not observed during the un-manipulated baseline study and caused a shift to a different magnetic response (quadramodal).

The experimental manipulation of symbiont density confirmed the findings of the baseline experiment. In the un-manipulated baseline experiment, crayfish with moderate densities of symbionts showed a strong magnetic alignment along the NE/SW axis and no significant topographic alignment, while crayfish with high symbiont densities did not exhibit consistent alignment relative to the magnetic field, but instead exhibited consistent alignment with respect to non-magnetic (topographic) cues. More specifically, alignments of crayfish with high symbiont densities were randomly distributed with respect to the magnetic field (but significantly aligned with respect to topographic cues), and the distribution of magnetic alignments in the moderate symbiont density treatment (Fig. 1C and see Fig. S1C) was significantly different from that in the high symbiont density treatment. In both experiments (i.e. manipulated and un-manipulated symbiont densities), increasing symbiont densities were associated with increased variability in the magnetic response of the hosts (Fig. 2, see Fig. S2 for topographic relationship). The 95% confidence interval around the observed baseline magnetic alignment axis did not include the direction of the animals’ native stream, nor the land-water axis, therefore suggesting an innate SMA behaviour rather than a response to the direction of the water flow or bank alignment at the capture site.

In the absence of symbiotic worms, crayfish changed their magnetic response, aligning quadramodally along the anti-cardinal magnetic directions (i.e. NE, SE, NW, SW) (Fig. 1B). Like the moderate density treatment, the crayfish without worms did not show consistent topographic alignment (see Fig. S1B).

**Figure 3:**
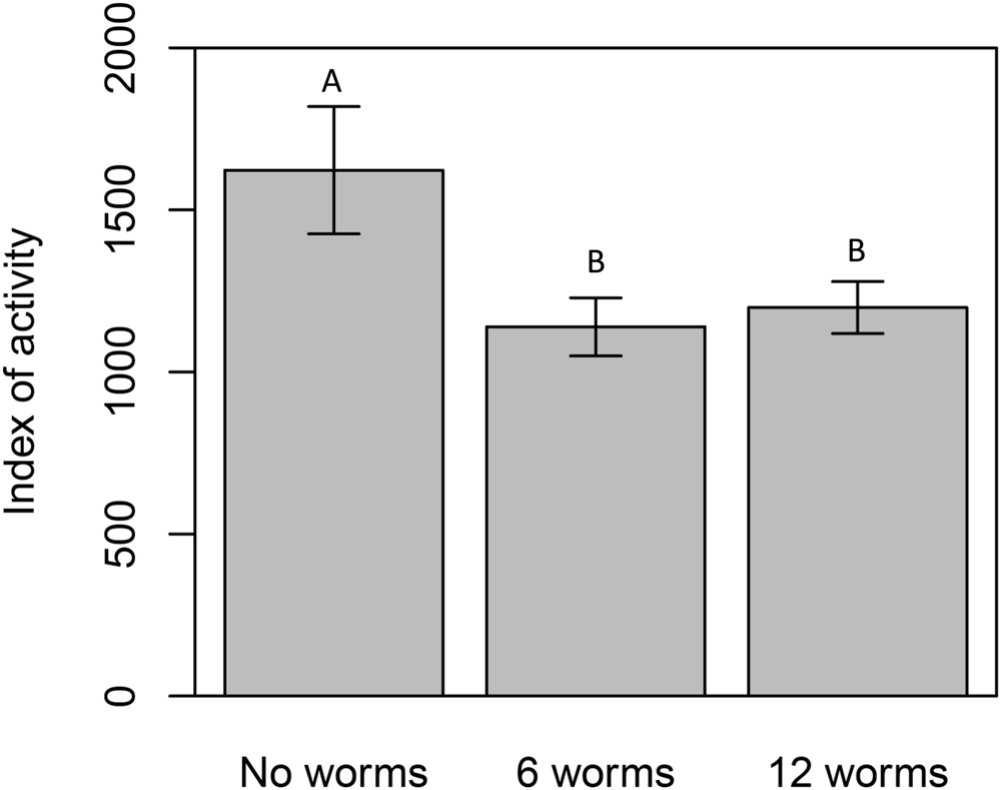
Activity analysis. Crayfish with moderate and high worm densities showed significantly lower activity than crayfish without any ectosymbionts (in the worm-density manipulation experiment).

The presence of branchiobdellidans also affected host locomotor behaviour. There was an overall significant effect of worm treatment (F_2,33_ = 9.2, p < 0.001), day of experiment (F_3,33_ = 6.488, p = 0.001), and a significant treatment by day interaction (F_6,33_ = 6.889, p < 0.001). Post hoc analysis showed a significant increase in host activity in the no-worm treatment, but no difference between moderate and high worm treatments (Fig. 3).

## Discussion

This study is the first study to demonstrate that crayfish can detect and respond to the Earth’s magnetic field. In baseline experiments with unmanipulated, natural ectosymbiotic worm densities, crayfish aligned axially along the NNE/SSW magnetic axis, closely resembling SMA responses in other animals, i.e., bimodal responses rotated slightly clockwise of the magnetic north-south axis^11,12,36–40^. Evidence from free-moving animals suggests that the NNE-SSW alignment is exhibited by both stationary animals (e.g., standing or grazing cattle and sleeping deer^41,42^), and by animals approaching a goal that is within their immediate field of view (e.g., foxes attacking rodent prey, or water birds landing on a water surface^36,39^). The relatively low level of activity of crayfish with moderate ectosymbionts densities that exhibited bimodal NNE-SSW magnetic orientation is consistent with spontaneous alignment being associated with proximity to (or occupation of) a fixed location (e.g., refuge); the residual motion of crayfish could reflect the inability of the crayfish to find cover in the experimental set-up.

One explanation that has been proposed for this type of spontaneous magnetic alignment stems from the possibility that the pattern of input from a light-dependent (photoreceptor-based) magnetic compass forms a 3-dimensional ‘visual’ pattern surrounding the animal that functions as a simple spherical grid or coordinate system^1,30^. This grid could help the animal to organize and structure spatial information from its surroundings (i.e., encode the relative positions of surrounding landmarks^1,30^) and/or magnetic input could be used to standardize visual input to the compound eye as shown in honeybees^9,10^. Standardizing the view of its surroundings by aligning itself along a fixed axis relative to the magnetic field might facilitate detection of novel targets that enter the crayfish’s field of view (predators, food items, rivals males, potential mates), analogous to ‘misplace cells’ in the rodent hippocampus^43^.

Crayfish without ectosymbionts exhibited distinctly different behaviour from those with moderate or high symbiont densities. Instead of aligning bimodally along the NNE-SSW magnetic axis, these crayfish aligned quadromodally along the anticardinal (NE-SE-SW-NW) directions relative to the magnetic field. Quadramodal (bi-axial) magnetic responses have been shown in a variety of both stationary and moving insects^44–48^. Interestingly, flies exhibit a form of systematic search using quadramodal movement patterns, i.e. 90° turn algorithms, when beaconing towards a food source is not possible^49,50^. Such search patterns have been shown mathematically to optimize search in taxonomically diverse animals^51–53^.

The role of magnetic cues in structured search behaviour of crayfish has not been investigated. Nevertheless, the increase in activity associated with the switch from bimodal to quadramodal SMA in crayfish without ectosymbionts could indicate a shift from a resting state to systematic search, perhaps using a ‘central place foraging’ strategy to systematically vary the directions of forays into the surrounding habitat^54^. The increased activity of crayfish in the no-worm treatment relative to both the medium and high-density groups is consistent with this hypothesis. It is conceivable that density-dependent effects of branchiobdellidans on host and symbiont fitness may select for host behaviour that increases the likelihood of dispersal/transmission from other hosts at zero worm densities. As discussed above, crayfish fitness is maximized at moderate worm densities while high densities are associated with a decrease in host growth and survival^22^. However, regular moulting (ecdysis) can greatly reduce ectosymbiont abundance/prevalence on crayfish^55^ and symbiont populations may need to be periodically replenished via horizontal transmission from other infested crayfish. Simultaneously, strong intra-symbiont competition at high densities decreases symbiont fitness^56^, which may lead to changes in branchiobdellidan feeding behaviours that result in damage to host tissues^26^. Because transmission of branchiobdellidans occurs mostly during direct physical contact among hosts^22^, increased movement and altered spatial behaviour could directly influence transmission rate by increasing host contact.

We speculate that the increased activity of crayfish without symbionts increases the likelihood of contact with other infested hosts that are a source of beneficial symbionts. Furthermore, the quadramodal magnetic alignment of crayfish without ectosymbionts coupled with the increase in activity is consistent with temporally and spatially structured behaviour underlying systematic search, as shown in flies^50^.

The observed change in relative salience of magnetic and non-magnetic cues at higher worm densities, i.e., densities that might be detrimental to the ectosymbionts^57^, as well as to their hosts, could result from host manipulation by the symbiont leading to increased transmission rates. At moderate densities, which are beneficial for symbiont and host^26,58^, crayfish tend to remain more aligned along the NNE-SSW axis, which has been associated with resting behaviour in other animals; see above^42^. One could speculate that crayfish burdened with high densities of symbionts might seek out refuges, and in these scenarios, reliance on non-magnetic (i.e., visual cues) may facilitate location of burrow entrances.

Symbioses are ubiquitous in nature. Given the importance of magnetic information in organizing spatial perception^59^, and the abundance of symbiont impacts on host behaviour, understanding how symbionts influence host responses to magnetic cues is likely to provide new insights into which factors may influence animal movements under natural conditions. It also advances our understanding of evolutionary forces that have shaped sensory systems that process spatial information, and, conversely, how symbiotic interactions influence various components of fitness in both hosts and ectosymbionts. In addition, understanding the effects of symbionts on host behaviour may help to account for some of the variability in the responses of wild caught host species to magnetic (and other) spatial cues. To the best of our knowledge, this is the first study to demonstrate that symbionts can alter their host’s behavioural response to the magnetic field, an effect that could be easily overlooked in un-manipulated field studies and in laboratory studies of wild caught host species. Our findings go beyond previous work showing that symbiosis involves complex interactions between the host and symbiont with possible fitness consequences for both taxa, suggesting that these interactions involve effects on host responses to a specific type of sensory input (i.e., magnetic cues) that play a fundamental role in organizing spatial behaviour.

## Supplementary information

Supplementary figures

Raw Data

## Data Availability

The datasets supporting this article have been uploaded as part of the supplementary material.
