# Supplementary material for: Ectosymbionts alter spontaneous responses to the Earth’s magnetic field in a crustacean
Source: Sci Rep. 2019 Feb 28;9:3105. doi: 10.1038/s41598-018-38404-7 (PMC6395607; doi:10.1038/s41598-018-38404-7)
Supplement: Supplementary file 1 — Supplementary figures [file 41598_2018_38404_MOESM1_ESM.pdf]

Ectosymbionts alter spontaneous responses to the Earth's magnetic field in a crustacean

Lukas Landler[1], James Skelton[1,2], Michael S. Painter[1, 3], Paul W. Youmans[1], Rachel Muheim[4], Robert P. Creed[5], Bryan L. Brown[1], John B. Phillips[1*]

[1] Department of Biological Sciences, Virginia Tech, Blacksburg, VA 24061, USA

[2]School of Forest Resources and Conservation, University of Florida, Gainesville, Florida 32603 USA

[3]Czech University of Life Sciences Prague, Department of Game Management and Wildlife Biology, Kamýcká 129, CZ - 165 21 Praha 6 - Suchdol, Czech Republic

[4]Department of Biology, Lund University, Lund, SE-221 00, Sweden

[5]Department of Biology, Appalachian State University, Boone, NC 28608, USA

***Corresponding author:** John B Phillips, Derring Hall RM 4100, 1405 Perry Street, Blacksburg, VA 24061, USA

Telephone number: +1 540 231 1481, email: jphillip@vt.edu

Supplementary information

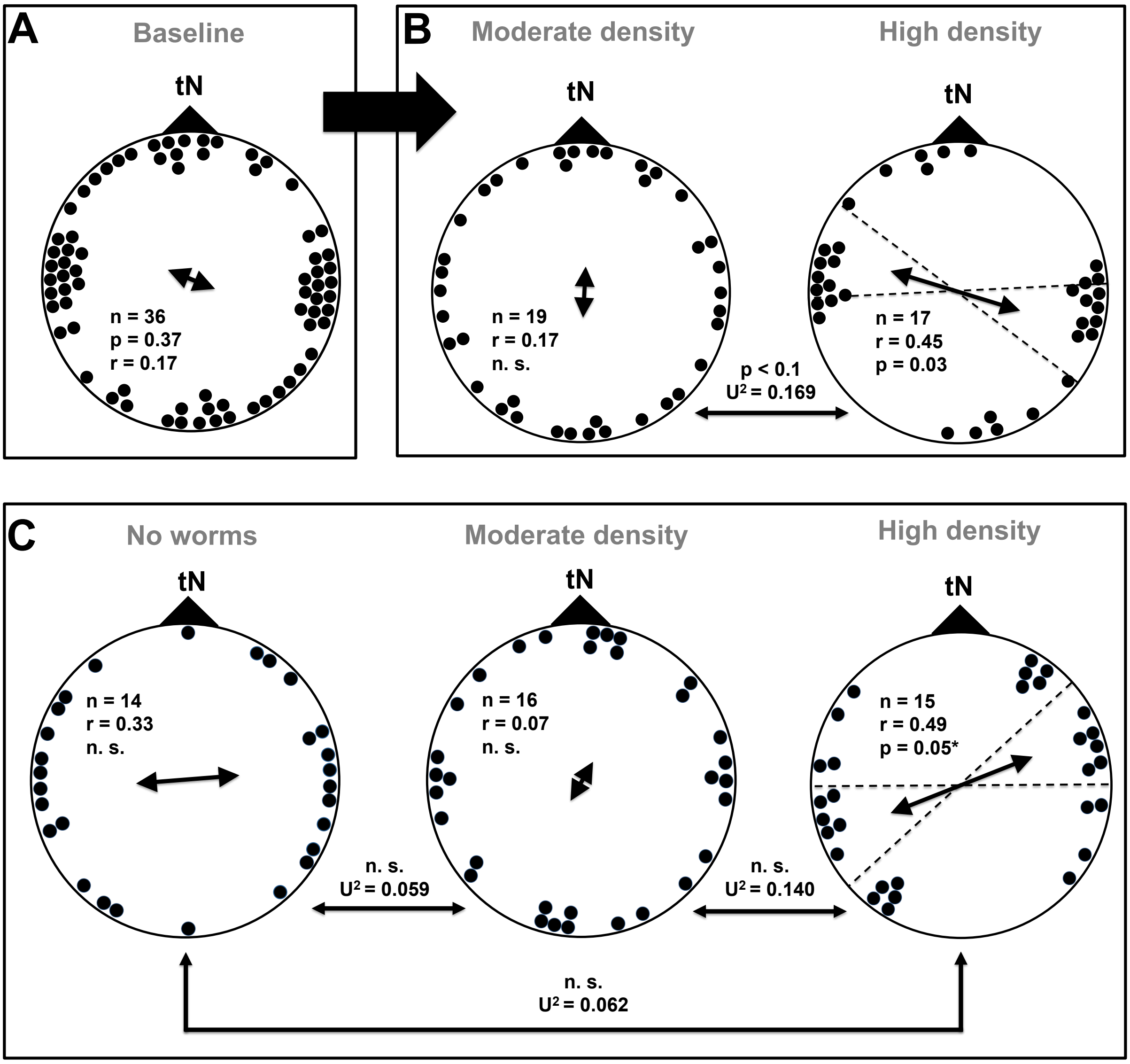

Figure S1: Topographic alignment of crayfish. a) The overall baseline test showed no significant alignment relative to the topographic north. **b)** Crayfish with high worm density (≥5 worms) showed significant topographic alignment, moderate worm density (<5 worms) crayfish were randomly aligned relative to topographic north. **c)** The experiment using three different ectosymbiont densities showed non-significant topographic alignment in the no worm treatment and in the moderate worm density (m) treatment. However, high worm density led to a significant topographic alignment. The exact directional cue used by the animals is not known, the set-up was as symmetrical as possible. However, the topographic

direction roughly corresponds with the side from which the experimenter entered the Faraday cage to introduce the animals at the start of the experiment. It is important to note, that the experimenter changed the magnetic fields from outside the Faraday cage and did not enter the experimental set-up during the recordings. All data are shown with axial data, no distinction between head and tail direction was made. Significance of alignments was tested by Rayleigh-test; p-value (p), sample size and the mean vector length (r) are given for each distribution. We compared distributions using the Watson U^2-test; test statistic (U^2) and p-values are shown above the arrows indicating the compared distributions. All p-values with an asterisk are alpha corrected, when tested for axial and bi-axial alignment. We calculated a 95% around the mean direction confidence interval in case of significance.

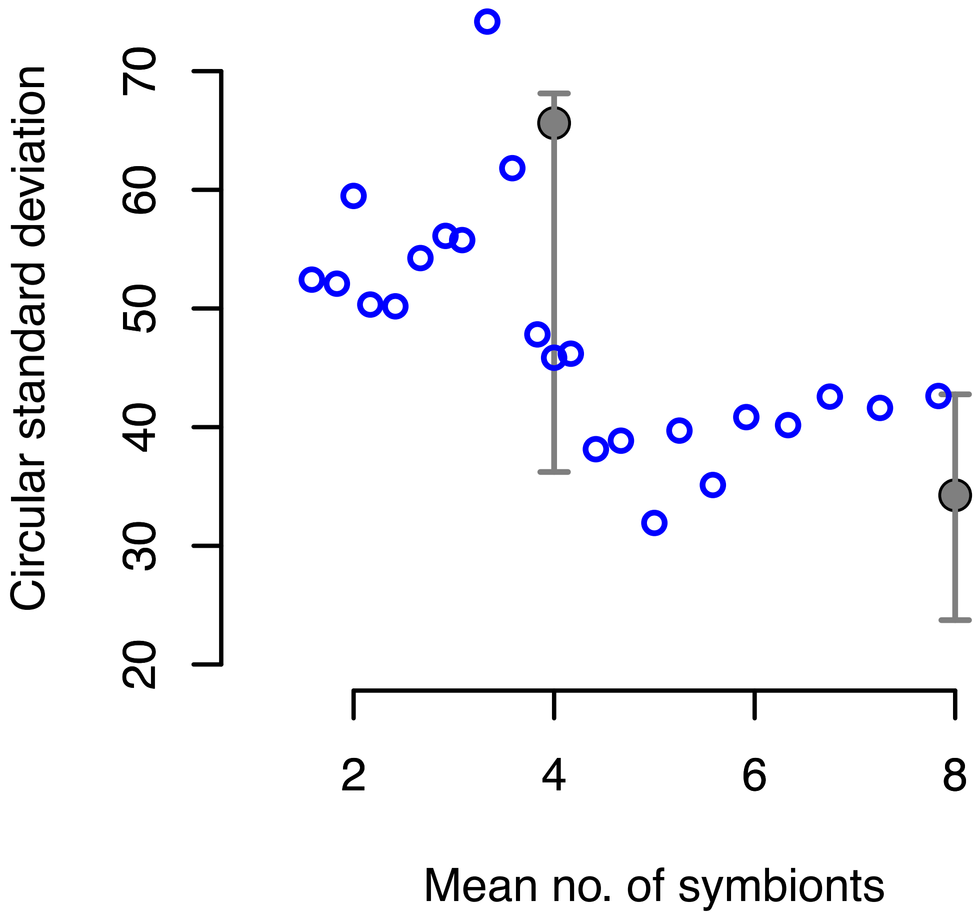

Figure S2: Topographic baseline response vs. worm manipulation experiment. Comparison of the relationship between variability of crayfish topographic alignment (circular standard deviation) and the number of symbionts during the baseline study with natural symbiont density (blue circles) and experimentally manipulated symbiont densities (grey circles). Variability in topographic orientation slightly decreased with increasing symbiont diversity in the baseline and manipulated experiments. Variability in baseline data calculated as the moving circular standard deviation of alignment direction across a moving window of twelve individuals sorted by observed natural symbiont density. Mean number of symbionts is the average number of *C. ingens* observed on the moving window of twelve

individuals. Variability for manipulated symbiont density is shown as the circular standard deviation for each treatment level. Error bars show 90% confidence interval base on bootstrapped values from 10,000 resamplings without replacement. Treatment levels were adjusted to reflect symbiont attrition prior to testing. The no-worm treatment is not shown; this condition was not observed during the un-manipulated baseline study and caused a shift to a different magnetic response (bi-axial), which would be misrepresented with an axial standard deviation.

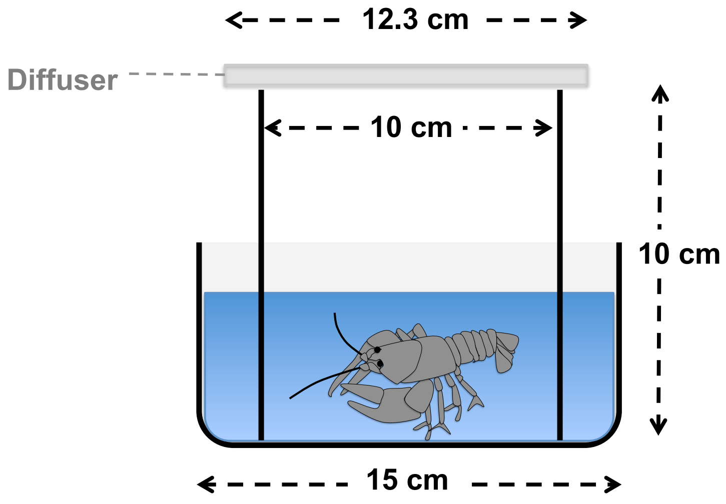

Figure S3: Experimental chamber. Crayfish were tested in visually symmetrical chambers, each one covered with a diffuser. Beneath each chamber a plastic mesh reflected the light and therefore prevented the animals from seeing the camera underneath.

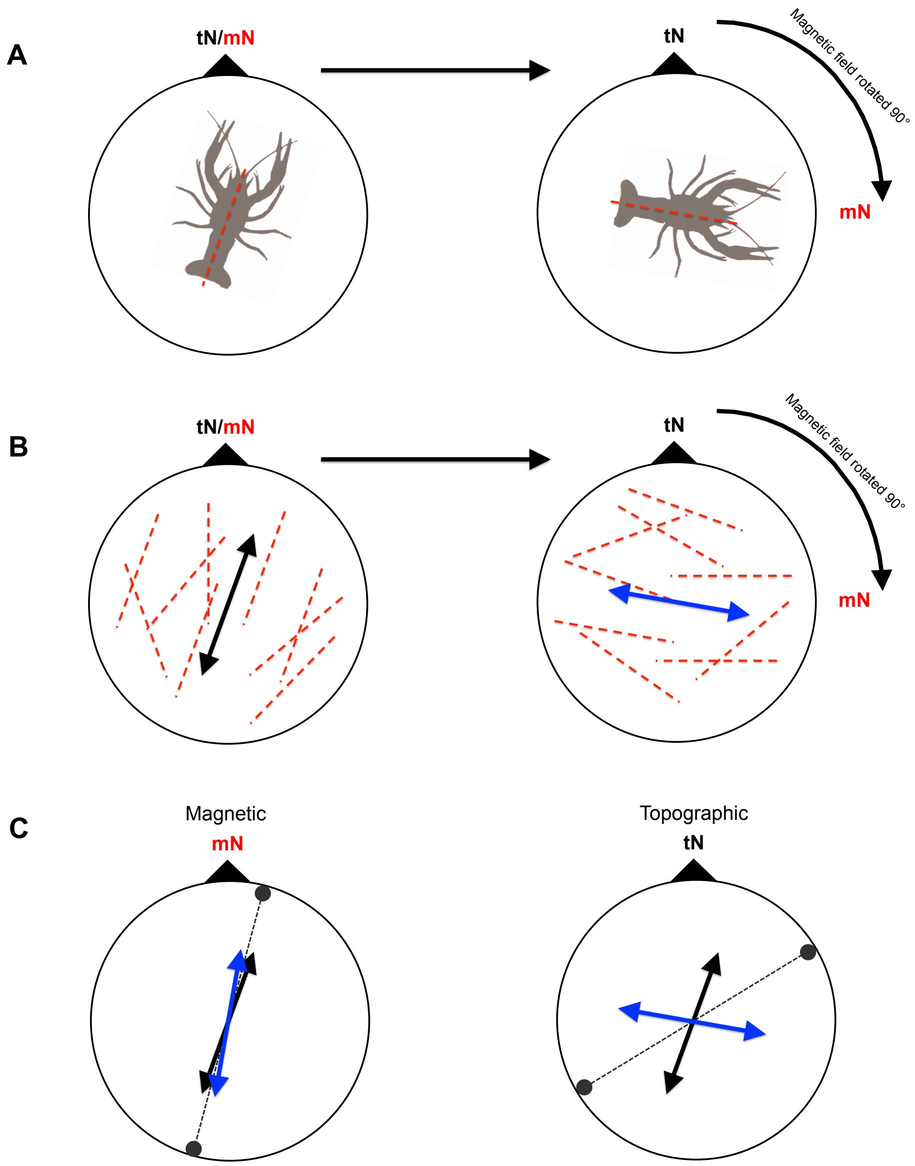

Figure S4: Methods used to de-couple magnetic and topographic crayfish alignment: a) Crayfish were tested singly in visually symmetrical chambers. Magnetic coils were used to alter the direction of simulated earth-strength magnetic north (mN) to all four cardinal topographic directions in randomized sequence. Shown is a rotation of mN from topographic north (left) to topographic east (right). **b)** Customized tracking software recorded the body axis orientation of crayfish (red dashed lines) during each trial at (1 s) intervals. For each crayfish under each magnetic field direction, the vector sum of all recorded orientations throughout the observation period were combined to generate a mean orientation vector. Shown are the mean orientation vectors when mN and tN are matched (black double-headed

arrow) and when mN is rotated 90° to topographic east (blue double-headed arrow). **c)** Mean orientation vectors for all four magnetic field directions, for each crayfish, were added and the resultant mean direction was taken as the axial mean bearing. Mean vectors were added as vectors relative to mN and vectors relative to tN, resulting in two different distributions, one showing the orientation in relation to the manipulated earth-strength magnetic north (left) and the other one showing the responses in relation to the unchanged topographic north (right).
